# l-Citrulline Protects from Kidney Damage in Type 1 Diabetic Mice

**DOI:** 10.3389/fimmu.2013.00480

**Published:** 2013-12-24

**Authors:** Maritza J. Romero, Lin Yao, Supriya Sridhar, Anil Bhatta, Huijuan Dou, Ganesan Ramesh, Michael W. Brands, David M. Pollock, Ruth B. Caldwell, Stephen D. Cederbaum, C. Alvin Head, Zsolt Bagi, Rudolf Lucas, Robert W. Caldwell

**Affiliations:** ^1^Department of Pharmacology and Toxicology, Georgia Regents University, Augusta, GA, USA; ^2^Department of Anesthesiology and Perioperative Medicine, Georgia Regents University, Augusta, GA, USA; ^3^Vascular Biology Center, Georgia Regents University, Augusta, GA, USA; ^4^Department of Medicine, Georgia Regents University, Augusta, GA, USA; ^5^Department of Physiology, Georgia Regents University, Augusta, GA, USA; ^6^Department of Cell Biology and Anatomy, Georgia Regents University, Augusta, GA, USA; ^7^Department of Ophthalmology, Georgia Regents University, Augusta, GA, USA; ^8^VA Medical Center, Georgia Regents University, Augusta, GA, USA; ^9^Intellectual and Developmental Disabilities Research Center/Neuropsychiatric Institute (IDDRC/NPI), University of California Los Angeles School of Medicine, Los Angeles, CA, USA; ^10^Division of Pulmonary Medicine, Georgia Regents University, Augusta, GA, USA

**Keywords:** arginase, l-citrulline, glomerulosclerosis, diabetic nephropathy, IL-10

## Abstract

**Rationale:** Diabetic nephropathy (DN) is a major cause of end-stage renal disease, associated with endothelial dysfunction. Chronic supplementation of l-arginine (l-arg), the substrate for endothelial nitric oxide synthase (eNOS), failed to improve vascular function. l-Citrulline (l-cit) supplementation not only increases l-arg synthesis, but also inhibits cytosolic arginase I, a competitor of eNOS for the use of l-arg, in the vasculature.

**Aims:** To investigate whether l-cit treatment reduces DN in streptozotocin (STZ)-induced type 1 diabetes (T1D) in mice and rats and to study its effects on arginase II (ArgII) function, the main renal isoform.

**Methods:** STZ-C57BL6 mice received l-cit or vehicle supplemented in the drinking water. For comparative analysis, diabetic ArgII knock out mice and l-cit-treated STZ-rats were evaluated.

**Results:** l-Citrulline exerted protective effects in kidneys of STZ-rats, and markedly reduced urinary albumin excretion, tubulo-interstitial fibrosis, and kidney hypertrophy, observed in untreated diabetic mice. Intriguingly, l-cit treatment was accompanied by a sustained elevation of tubular ArgII at 16 weeks and significantly enhanced plasma levels of the anti-inflammatory cytokine IL-10. Diabetic ArgII knock out mice showed greater blood urea nitrogen levels, hypertrophy, and dilated tubules than diabetic wild type (WT) mice. Despite a marked reduction in collagen deposition in ArgII knock out mice, their albuminuria was not significantly different from diabetic WT animals. l-Cit also restored nitric oxide/reactive oxygen species balance and barrier function in high glucose-treated monolayers of human glomerular endothelial cells. Moreover, l-cit also has the ability to establish an anti-inflammatory profile, characterized by increased IL-10 and reduced IL-1β and IL-12(p70) generation in the human proximal tubular cells.

**Conclusion:** l-Citrulline supplementation established an anti-inflammatory profile and significantly preserved the nephron function during T1D.

## Introduction

Patients with Type 1 diabetes (T1D) have a considerably worse long-term prognosis than individuals without diabetes, due to the high incidence of cardiovascular disease and end-stage renal disease (ESRD). Diabetic nephropathy (DN), the leading cause of chronic kidney disease in the United States, is responsible for up to 40% of all ESRD cases (1). Since conventional or recently proposed therapies toward DN are still under ongoing investigation, or lack major efficacy, the search for novel targets involved in diabetes-induced renal damage is of primary importance.

It is now generally recognized that dysfunction of endothelial nitric oxide synthase (eNOS) contributes to vascular pathology in diabetes. An important cause of impaired endothelial nitric oxide (NO) production is the reduced availability of the eNOS substrate l-arginine (l-arg). Patients with diabetes and cardiovascular disease were shown to benefit from acute l-arg supplementation (2), but chronic l-arg therapy caused adverse effects (3).

Oral l-citrulline (l-cit, precursor of l-arg) increases circulating levels of l-arg and augments NO-dependent signaling (4, 5), not only by means of increasing l-arg synthesis but also by decreasing l-arg catabolism (6). The latter activity occurs due to l-cit’s capacity to allosterically inhibit arginase I (ArgI), an enzyme which can impair eNOS function (7, 8). As such, this dual effect of l-cit makes it a suitable supplemental amino acid to provide sufficient l-arg for proper eNOS function. In this regard, l-cit has been shown to prevent coronary vascular dysfunction in diabetic rats (8), with concomitant reduction of endothelial ArgI activity, which was also recently shown to contribute to coronary endothelial dysfunction in patients with diabetes mellitus (9) and in diabetic mice (10).

The effects of l-cit on vascular endothelial function may also positively influence the endothelial glycocalyx, thus contributing to glomerular barrier preservation (11, 12). However, l-cit supplementation has been neither evaluated in a model of diabetic kidney disease, nor its effects on renal arginase. In the kidneys, arginase II (ArgII) is the only isoform expressed in mouse and humans (13). ArgII is present in the proximal tubules (PT) and in the inner medullary collecting ducts (14) and plays an important role in renal physiology and homeostasis (15). Arginase metabolizes l-arg to urea and ornithine. Whereas urea has a key role in the urinary concentrating mechanism (16), ornithine is the substrate for the ornithine/polyamine and ornithine/proline pathways. Both of these pathways play an important role in kidney physiology and pathology (17–19). Indeed, production of polyamines enhances progression of the cell cycle and is associated with cell survival (20). Proline, on the other hand, is a precursor needed for collagen synthesis (21). Thus, although these mechanisms are important to maintain kidney function, they may also contribute to kidney hypertrophy and glomerulosclerosis of diabetes. Up-regulation of renal ArgII, proposed to be a mediator of DN, may play a role in these processes (22). However, l-cit supplementation to newborn rats was accompanied by enhanced ArgII expression in lungs, but it still protected from pulmonary hypertension (23).

In this study, we determined whether l-cit supplementation to streptozotocin (STZ)-diabetic rodents blunts the development of DN, and whether l-cit has an effect on renal ArgII.

## Materials and Methods

### Animals and diabetic model

Experiments were performed with C57BL/6 wild type (WT) mice (Jackson Laboratories, Bar Harbor, ME, USA), or ArgII homozygous knockout mice on a C57BL/6 background (24, 25). Ten-week old male mice (18–20 g) were rendered diabetic with intraperitoneal injections of STZ (65 mg/kg) (Sigma Aldrich, St. Louis, MO, USA), on alternating days for up to four injections (10). A group of control (vehicle) and diabetic mice were treated with l-cit (50 mg kg^-1^ day^-1^, supplemented in drinking water) (8). Animals were housed in individual cages. The l-cit dose was adjusted to each animal according to the daily water intake. Mice were studied after 2 and 16 weeks with diabetes. In addition, male Sprague-Dawley rats (Charles River Laboratories, Raleigh, NC, USA), weighing between 225 and 250 g, were rendered diabetic with a single dose of STZ (50 mg/kg, intraperitoneally). A group of diabetic rats (≥350 mg/dl) was treated with l-cit, as indicated above. Rats were studied after 8 weeks with diabetes. Animals had free access to food and water throughout the study. All animals received humane care in compliance with federal laws and institutional guidelines at Georgia Regents University.

### Measurement of kidney hypertrophy

Determination of kidney to body weight ratio was used as a measure of kidney hypertrophy. The left kidney was removed, decapsulated, placed on tissue paper for 1 min, and weighed.

### Analytical methods

Mouse urinary albumin excretion (UAE), and rat proteinuria were determined after 24 h urine collection, using an ELISA kit (AssayPro, St. Charles, MO, USA), and a protein assay kit (BCA Pierce, Rockford, IL, USA), respectively. Blood glucose levels were measured by the Alpha Trak-Blood glucose monitoring system (Abbott Laboratories, St. Clara, CA, USA). Plasma urea levels were measured by colorimetric determination of urea at 540 nm in the presence of α-Isonitrosopropiophenone (α-ISPF, 9% in ethanol) (Sigma Aldrich, St. Louis, MO, USA). Results were expressed as milligram per deciliter of blood urea nitrogen (BUN). Mouse plasma samples, separated from heparinized whole-blood, were used for the measurement of 32 cytokines and chemokines, using a magnetic bead-based multiplex assay, as described in Ref. (26) (32 Multiplex MCYTOMAG-70K assay, EMD Millipore).

### Tissue histology

After being excised and decapsulated, mouse kidneys were immersed in 10% formalin for 24 h, embedded in paraffin and sectioned at 4 μm thickness. Sections were deparaffinized in xylene, rehydrated through graded ethanols to water, and stained with periodic acid Schiff (PAS) for morphology evaluation. Picro-Sirius red was used to stain for tissue collagen. Rat kidneys were frozen in liquid nitrogen, and cryosections (5 μm) were air-dried for 30 min. Cryosections stained with Picro-sirius red were processed as previously described in Ref. (27). All PAS and Picro-sirius red-stained sections were visualized on a computer connected to a light microscope (AxioVision; Carl Zeiss Meditec, Inc.). Quantitative analysis of collagen was performed on photomicrographs of kidney sections by using specific software (Image J). Seven to ten non-overlapping fields per section were analyzed for each animal. Tissue collagen content was assessed by a fibrosis index (%) that indicated the ratio of the mean sirius red-stained area to the mean whole area of the section, calculated as the mean of the fibrosis indexes for each section for each animal.

### Renal arginase activity

Renal arginase activity (RAA) was measured in kidney cortex homogenized in ice-cold lysis buffer (50 mmol/L Tris-HCl, 0.1 mmol/L EDTA and EGTA, pH 7.5) at 1:4 (wt:vol) ratio, containing protease inhibitors. The homogenate was centrifuged at 14,000 × *g* for 20 min. The supernatant was removed for enzyme assay using a colorimetric determination of urea production from l-arg, as previously described in Ref. (28). Samples were assayed in triplicate. Values were corrected by adjusting for protein concentration in the homogenate and expressed as nanomole urea per milligram protein per hour. Additional corrections were made after subtracting basal levels of urea obtained from each sample of kidney cortex homogenates in the absence of MnCl_2_ and of l-arg.

### Western blot analysis

Mouse and rat frozen kidney cortex were pulverized and homogenized in RIPA lysis buffer (EMD Millipore, Billerica, MA, USA), containing protease and phosphatase inhibitor cocktails (Sigma Aldrich, St. Louis, MO, USA). Soluble protein extracts from tissue homogenates were subjected to SDS-PAGE electrophoresis, transferred to polyvinylidene fluoride membranes and reacted with anti-ArgII primary antibody (1:500, Santa Cruz Biotechnology, St. Cruz, CA, USA), at 4°C overnight. Subsequently, the bound antibody was detected by donkey anti-rabbit horseradish peroxidase-labeled secondary antibody (1:6,000, GE Healthcare, Pittsburgh, PA, USA), and visualized with ECL substrate (Amersham, Buckinghamshire, UK). Membranes were then stripped and re-probed with anti-GAPDH (Santa Cruz Biotechnology, St. Cruz, CA, USA) to assess level of protein loading. Protein expression was determined using densitometry analysis of films.

### Immunohistochemistry

Immunohistochemical detection of ArgII was performed in deparaffinized and rehydrated mouse kidney sections by means of light microscopy studies. Briefly, antigen retrieval was performed by immersing the slides in 0.01 M citrate buffer (pH 6.0), at 95°C for 30 min in a water bath. Endogenous biotin and peroxidase activity were blocked before staining, by using commercial avidin/biotin and peroxidase kits, respectively (Vector Laboratories, Burlingame, CA, USA). Slides were then incubated for 1 h with primary antibody against ArgII (1:500). The primary antibody was localized using the VECTASTAIN ABC-Elite peroxidase detection system (Vector Laboratories, Burlingame, CA, USA). Primary antibody against kidney injury molecule 1 (KIM-1) (1:500, R&D Systems, Minneapolis, MN, USA), followed by anti-goat secondary antibody (1:6,000, Invitrogen, Grand Island, NY, USA), were used for immunofluorescent staining of rat frozen sections. Nuclei were counterstained with DAPI. All sections were examined by two different researchers in a blinded manner. The number of tubules that exhibited positive red fluorescent staining to KIM-1 was counted per field. Five to seven fields were examined in each kidney section. Sections of each kidney were processed in parallel with the appropriate negative control tissue, processed with omission of the primary antibody in the staining procedure.

### Human glomerular endothelial cell culture

Human glomerular endothelial cells (Lonza, Walkersville, MD, USA) were grown in complete CSC medium, and maintained at 37°C in a humidified 5% CO_2_ incubator. Cells were used between passages four and six for the experiments. Treatment of cells with normal (5.5 mM, NG) or high [25 mM, high glucose (HG)] d-glucose-supplemented medium was performed in basic CSC medium. As control for the osmotic effect of high d-glucose, l-glucose was added to the basic endothelial medium. Pre-treatment of HGEC with l-cit (1 mM) was performed by adding the amino acid 2 h prior to adding HG or iso-osmotic control. HGEC were cultured under NG or HG conditions for either 24 h or 14 days, before they were used for experiments.

### Mitochondrial superoxide

Human glomerular endothelial cells were seeded in 0.2% gelatin-coated four well slide chambers at 1 × 10^5^ cells per well, and allowed to reach confluence. Then cells were exposed to HG for 24 h as described above, with or without pre-treatment with l-cit. At the end of incubation, MitoSOX (Invitrogen) 5.0 μM was added to the cells and incubated further for 10 min at 37°C in 5% CO_2_ atmosphere, according to manufacturing instructions. Subsequently, cells were washed in hanks balanced salt solution (HBSS, with Ca/Mg) and used for confocal microscopy imaging. The digital images were taken by an inverted confocal laser scanning microscope LSM Pascal (Zeiss, Germany), with an excitation/emission of 510/580 nm. Images were captured using 40× oil immersion objective lens.

### Nitric oxide metabolite

Human glomerular endothelial cell were seeded at 1 × 10^5^ cells per well in 24-well plates. Confluent quiescent cell monolayers were exposed to HG or proper iso-osmotic control for 24 h. l-Cit (1 mM) was applied 2 h prior to HG. Exposure was terminated by removal of the supernatant. Fresh basic CSC medium was replaced and cells incubated for additional 30 min. Supernatant was then removed, subsequently centrifuged and stored at −80°C for NO analysis. Cell supernatants containing nitrite $\left({\mathrm{NO}}_{2}^{-}\right)$ the stable breakdown product of NO in aqueous medium were refluxed in glacial acetic acid containing sodium iodide. ${\mathrm{NO}}_{2}^{-}$ is quantitatively reduced to NO under these conditions, which can be quantified by a chemiluminescence detector in a NO analyzer (Sievers) as described in Ref. (8).

### Permeability assay of HGEC monolayers

Human glomerular endothelial cell monolayer permeability to high molecular mass proteins was assayed by using 2,000-kDa FITC-dextran, based on the Transwell model (EMD Millipore). For this, HGEC were seeded on collagen-coated Transwells at a density of 1 × 10^5^ cells per well in 250 μl of CSC growth medium. The inserts were placed into 24-well plates containing 500 μl of medium. Upon reaching confluence, HGEC were exposed to HG as described above, with or without pre-treatment with l-cit. Transendothelial passage of dextran was determined after 14 days of incubation in HG media as described previously (12). Briefly, medium was aspirated and 150 μl of FITC-dextran was added into the insert and incubated for 3 h. The insert was then removed, and 100 μl of medium was collected from the bottom chamber and transferred to a black 96-well plate. The fluorescent density of samples was analyzed on a Paradigm Microplate Fluorometer (Beckman-Coulter) at 485 nm excitation and 530 nm emission wavelengths.

### Human proximal tubular epithelial cell culture

Human proximal tubular epithelial cell (huPTEC) (Lifeline Cell Technology, Frederick, MD, USA) were grown in the commercial RenaLife medium, and maintained at 37°C in a humidified 5% CO_2_ incubator. Cells were used between passages two and four for the experiments. Treatment of cells with normal (5.5 mM, NG) or high (25 mM, HG) d-glucose-supplemented medium was performed in six-well plates and maintained for 7 days, before they were used for experiments. As control for the osmotic effect of high d-glucose, l-glucose was added to the culture medium. Pre-treatment of huPTEC with l-cit (1 mM) was performed by adding the amino acid 2 h prior to adding HG or iso-osmotic control, with or without concurrent pre-treatment with a neutralizing anti-human IL-10 antibody (5 μg/ml, R&D Systems). Upon completion of treatment, culture medium supernatants were collected, centrifuged, and freeze at −80°C until use for cytokine measurement. Cells were lysed in RIPA buffer and protein extracts were loaded for Western blot analysis of ArgII as described for tissue extracts.

### Multiplex human cytokine/chemokine measurement

A panel of 13 pro-inflammatory cytokines [interferon-γ (IFN-γ), IL-1β, IL-2, IL-4, IL-5, IL-6, IL-7, IL-8, IL-10, IL-12p70, IL-13, TNF, and granulocyte-monocyte colony-stimulating factor (GM-CSF)] was assessed in triplicates in 50 μl cultured medium supernatants from cultured primary huPTEC, using a highly sensitive magnetic beads-based kit (MILLIPLEX MAP High Sensitivity Human Cytokine Panel – Premixed 13 Plex, EMD Millipore) (29). This assay has a high sensitivity, typically with a detection limit in the range from 0.01 to 0.48 ng/l.

### Immunofluorescence staining of cultured huPTEC

Cells were seeded in slide chambers at 1 × 10^5^ cells per well. When cells reached about 75–80% confluence, HG was added for 1 week as described above, with or without pre-treatment with l-cit. Upon completion of treatment, cells were washed twice with PBS and fixed with 4% paraformaldehyde for 15 min. Then, a blocking solution (1X PBS/5% normal goat serum/0.3% Triton™ X-100) was applied to the attached cells in the slide chambers for 1 h, prior to addition of anti-caspase 6 antibody (1:800, Cell Signaling, Boston, MA, USA) for incubation overnight at 4°C. Cells were washed twice with PBS and incubated with a fluorochrome-conjugated secondary antibody (1:400, Cy5 goat anti-rabbit, Jackson ImmunoResearch). DAPI was used for nuclear staining. For non-specific binding (negative control) the primary antibody was omitted. Images were collected with fluorescent microscopy. Fluorescence intensity measurements were performed in nuclei, normalized to DAPI nuclei area, and corrected by subtraction of background from negative controls.

### Statistical analysis

All data were expressed as mean ± SEM. Statistical analysis was performed by one-way ANOVA with a Tukey post test. In some experiments, statistical differences were determined by a Student’s *t*-test. A *p* value of <0.05 was considered statistically significant.

## Results

### Blood glucose, water consumption, urine volume, body weight, kidney weight, and BUN in mice

All diabetic groups had elevated blood glucose levels and increased daily water intake and urinary volume excretion, both at 2 (Table 1) and 16 weeks (Table 2) of the disease vs. respective non-diabetic controls. The kidney hypertrophy and wasting of body mass detected in untreated diabetic WT mice was not observed in l-cit-treated mice, despite significant hyperglycemia (Tables 1 and 2). Intriguingly, although ArgII has been proposed to be a mediator of DN (22), we observed a significant greater kidney size and BUN levels in the ArgII knock out mice, as compared to diabetic WT animals (Table 2). These results indicate that l-cit does not affect blood glucose levels in the diabetic state, but prevents body weight loss and kidney hypertrophy. In addition, the results observed in the ArgII knock out mice suggest that the lack of ArgII enhances diabetes-induced kidney hypertrophy and may accelerate the decay of kidney function in diabetic mice.

**Table 1:** **Biochemical and physical characteristics of study groups after 2 weeks. Effect of l-cit supplementation**.

	Blood glucose (mg/dl)	Water intake (ml/day)	Urine volume (ml/day)	Body weight (g)	K/BW ratio
Control	103.8 ± 8.2	7 ± 0.9	1.55 ± 0.2	23 ± 0.7	5.97 ± 0.4
Diabetic	460.3 ± 71.7^a^	19.5 ± 2.4^b^	14.5 ± 1.4^c^	19 ± 0.5^d^	8.53 ± 0.03^d^
l-Cit-Con	132.3 ± 11.1	7.2 ± 0.6	1.4 ± 0.4	26 ± 1.3	6.39 ± 0.4
l-Cit-Diab	465.8 ± 96.4^a^	17.5 ± 1.1^b^	9.6 ± 2.4^c^	25 ± 0.4	5.85 ± 0.1

*K/BW, kidney/body weight ratio; control, untreated control mice; diabetic, untreated diabetic mice; l-cit-Con, l-cit-treated control mice; l-cit-Diab, l-cit-treated diabetic mice. Values are expressed as mean ± SEM*.

*^a^*p* < 0.001 vs. control groups; ^b^*p* < 0.01 vs. control groups; ^c^*p* < 0.05 vs. control groups; ^d^*p* < 0.01 vs. all groups*.

**Table 2:** **Biochemical and physical characteristics of study groups after 16 weeks. Effect of l-cit supplementation and ArgII deletion**.

	Blood glucose (mg/dl)	Water intake (ml/day)	Urine volume (ml/day)	Body weight (g)	K/BW ratio	BUN (mg/dl)
Control	148.8 ± 20.1	7.3 ± 1.1	2.2 ± 0.3	28 ± 0.3	6.80 ± 0.5	10.52 ± 0.5
Diabetic	546.2 ± 19.7^a^	19.3 ± 2.2^a^	19.2 ± 1.7^c^	21 ± 0.2^d^	9 ± 0.4^g^	12.03 ± 0.5
l-Cit-Con	119 ± 6.8	7.2 ± 0.6	1.6 ± 0.3	31 ± 0.8^f^	6.86 ± 0.3	14.24 ± 0.8
l-Cit-Diab	518.3 ± 27^a^	26.8 ± 2.1^b^	20.9 ± 2.3^c^	26 ± 0.5^e^	6.93 ± 0.4	13.36 ± 0.33
C ArgII KO	133.7 ± 18.9	6.9 ± 0.5	2 ± 0.3	21 ± 1.8	8.13 ± 0.3^h^	15 ± 2.5
D ArgII KO	554.8 ± 54.5^a^	30.3 ± 3.6^b^	28.2 ± 1.9^c^	20 ± 0.6	11.45 ± 0.8^i^	17.07 ± 1.3^b^

*K/BW, kidney/body weight ratio; BUN, blood urea nitrogen; control, untreated control WT mice; diabetic, untreated diabetic WT mice; l-cit-Con, l-cit-treated control WT mice; l-cit-Diab, l-cit-treated diabetic WT mice; C ArgII KO, control ArgII knock out mice; D ArgII KO, diabetic ArgII knock out mice. Values are expressed as mean ± SEM*.

*^a^*p* < 0.01 vs. all control groups; ^b^*p* < 0.05 vs. all groups; ^c^*p* < 0.05 vs. all control groups; ^d^*p* < 0.001 vs. control and l-cit-Con; ^e^*p* < 0.001 vs. diabetic; ^f^*p* < 0.001 vs. l-cit-Diab; ^g^*p* < 0.01 vs. control and Lcit-Con; ^h^*p* < 0.05 vs. control; ^i^*p* < 0.01 vs. all groups*.

### Renal arginase activity and ArgII protein levels

**Figure 1:**
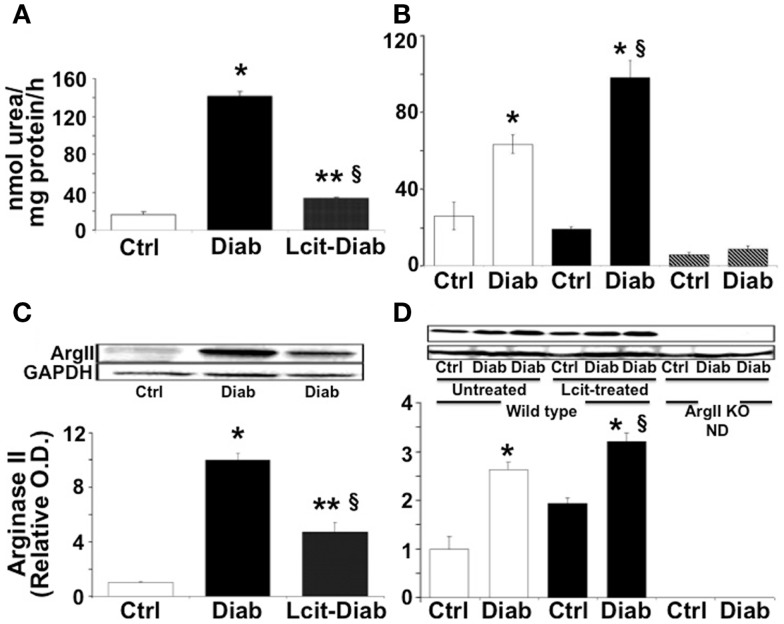
**Renal arginase**. Kidney cortex isolated from mice after 2 **(A–C)** and 16 **(B–D)** weeks of diabetes was homogenized in lysis buffer. Arginase activity **(A,B)** was assayed using a colorimetric determination of urea production from l-arginine. Relative levels of arginase II protein expression **(C,D)** were determined by western blot analysis. Ctrl, control; Diab, diabetic; WT, wild type; ArgII KO, ArgII knock out; ND, not detected. Values are expressed as mean ± SEM, *n* = 4–6. **(A–C)** *p < 0.001 vs. Ctrl, ^§^*p* < 0.01 vs. Ctrl, **p < 0.001 vs. Diab. **(B–D)** 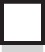, Untreated WT; 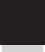, l-cit-treated WT; 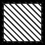, ArgII KO. *p < 0.01 vs. untreated and l-cit-treated Ctrl WT, and ArgII KO groups, ^§^*p* < 0.05 vs. untreated Diab WT.

At 2 weeks, RAA was elevated in untreated diabetic WT mice by 8.6-fold over control. By contrast, l-cit-treated diabetic WT mice showed only twofold elevated RAA levels over control values (Figure 1A). The marked elevation of RAA, observed at 2 weeks in untreated diabetic WT mice, declined by 16 weeks to a level of ~2.4-fold over respective control. At that time period, diabetic WT mice treated with l-cit showed a rise in RAA of 3.8-fold over control (Figure 1B). These results indicate that diabetes strongly induces arginase activity in renal tissues, and that long-term supplementation of l-cit does not prevent this effect. The absence of the ArgII gene in both control and diabetic ArgII knock out mice, resulted in RAA values below control WT mice by 0.2- and 0.3-fold, respectively. These low levels of arginase activity could be due to the presence of vascular and blood cell-derived ArgI.

Western blot analysis of protein extracts from kidney cortex homogenates of untreated diabetic WT mice showed levels of ArgII protein that were increased up to 10-fold over control at 2 weeks (Figure 1C). ArgII protein in tissues from l-cit-treated diabetic WT mice were ~fivefold higher than in controls (Figure 1C). Conversely, upon progression of diabetes to 16 weeks, the highest levels of ArgII protein were observed in l-cit-treated diabetic WT mice (Figure 1D). ArgII was neither detected in control nor in diabetic ArgII knock out mice (Figure 1D). These results indicate that the induction of arginase activity observed in kidney cortex of diabetic mice is due to increased protein levels of ArgII. l-Cit does not prevent diabetes-induced ArgII up-regulation, and may even have an additive effect upon long-term supplementation.

### Immunohistochemistry

**Figure 2:**
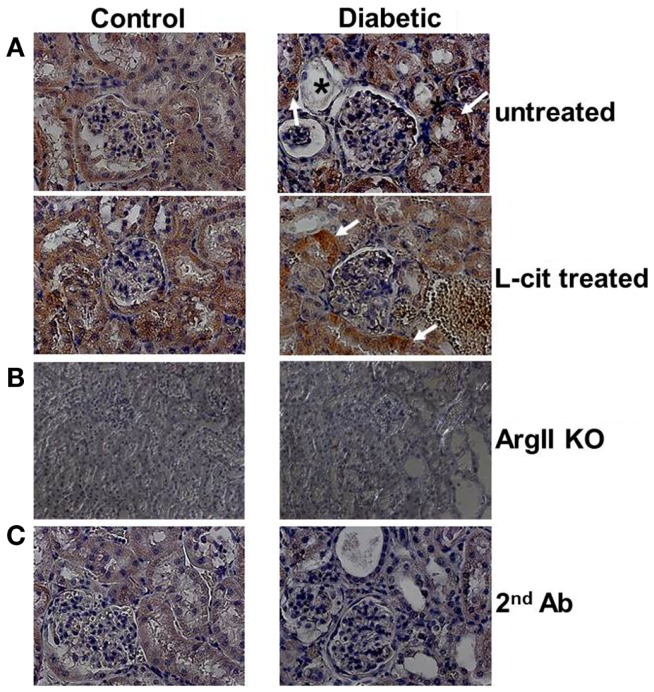
**Immunohistochemistry of ArgII in paraffin-embedded mouse kidney sections**. Representative photographs of paraffin-embedded kidney sections showing immunolocalization of ArgII (brown color; 40× immersion oil). **(A)** Cortical tubular segments in untreated diabetic mice show higher stain density than untreated control mice, after 16 weeks (white arrows). Atrophic tubules with widened lumina and flattened tubular cells, or with altered epithelial morphology, show reduced ArgII staining (*). l-Cit-treated diabetic mice show also enhanced ArgII stain intensity (white arrows). Kidney segments of control and diabetic ArgII knock out mice **(B)**, or tubules stained with secondary antibody alone **(C)** were completely negative for ArgII immunostaining.

Diffuse ArgII immunoreactivity was observed in cells of the urinary pole of the Bowman’s capsule, and of the PT of untreated control WT mouse kidneys. Enhanced tubular ArgII staining was detected in untreated diabetic WT mice after 16 weeks of the disease (Figure 2A). l-Cit-treated diabetic WT mice also demonstrated increased ArgII immunoreactivity in cortical tubular segments, while maintaining a more conserved epithelial morphology (Figure 2A). No positive staining was observed in either the ArgII knock out mouse kidneys (Figure 2B), or in tissue sections stained in parallel with omission of primary antibody (Figure 2C).

### Urinary albumin excretion

**Figure 3:**
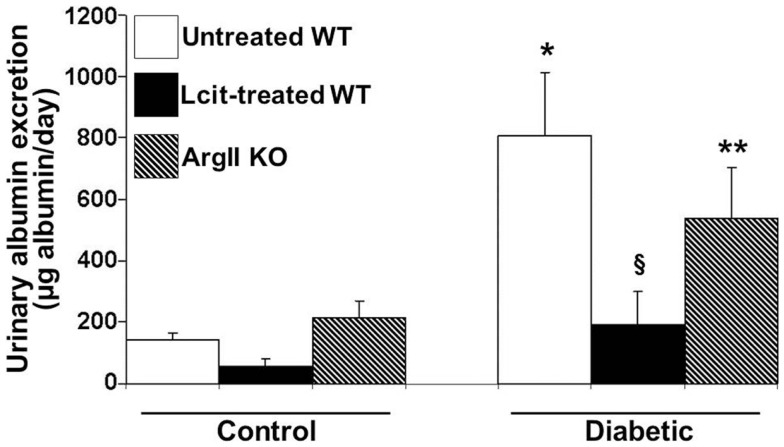
**Urinary albumin excretion (UAE) in mice after 16 weeks**. Mice were placed in metabolic cages. Urine samples were collected for 24 h to determine albumin excretion. Values are expressed as mean ± SEM, *n* = 5–9. WT, wild type; ArgII KO, ArgII knock out. *p < 0.001 vs. untreated, l-cit-treated, and ArgII KO control, ^§^*p* < 0.05 vs. untreated diabetic WT, **p < 0.05 vs. untreated and l-cit-treated control WT.

Urinary albumin excretion was significantly elevated above control in untreated diabetic WT mice as early as 2 weeks, but this effect was markedly blunted upon l-cit treatment (untreated diabetic: 811.43 ± 161.04 μg/ml vs. control: 97.73 ± 29.6 μg/ml, and l-cit-treated diabetic: 138.47 ± 47.3 μg/ml, *p* < 0.05). This preventive effect of l-cit on urinary albumin leakage was observed for up to 16 weeks, while non-treated diabetic WT mice maintained elevated UAE at that time point (Figure 3). Urine samples from diabetic ArgII knock out mice showed a trend to reduced albumin excretion, as compared to non-treated diabetic WT mice (Figure 3). These data thus indicate that l-cit may be protective toward diabetes-induced glomerular barrier dysfunction and/or impairment of proximal tubular protein uptake.

### Renal histology

**Figure 4:**
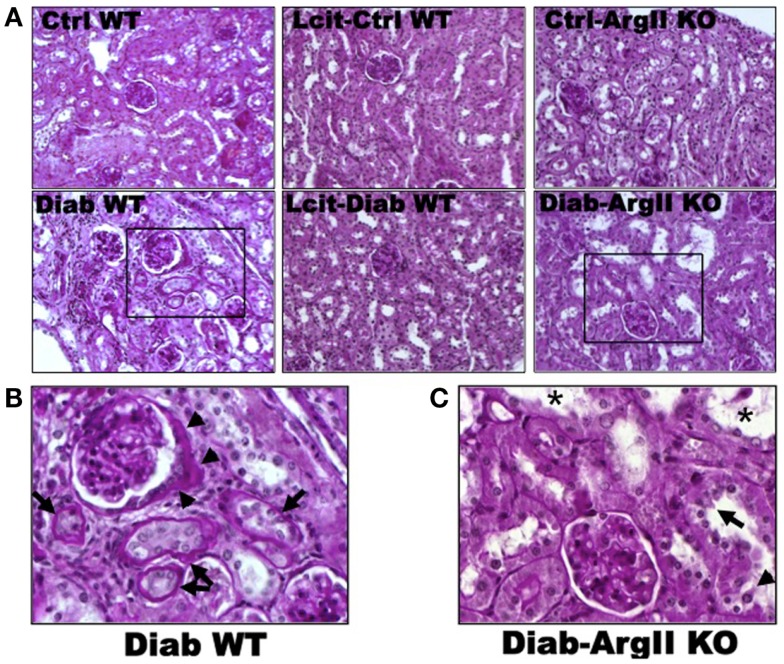
**Periodic acid Schiff-stained paraffin-embedded kidney sections**. **(A)** Representative photomicrographs of PAS-stained kidney sections from: untreated (Cont WT) and l-cit-treated (Lcit-Cont WT) control wild type, and control ArgII knock out (Ctrl-ArgII KO) mice (upper). Untreated (Diab WT) and l-cit-treated (Lcit-Diab WT) diabetic wild type, and diabetic ArgII knock out (Diab-ArgII KO) mice (lower) at 16 weeks (20×). Morphological alterations of a selected area (rectangle) observed in Diab WT and Diab-ArgII KO kidneys are shown at higher magnification (40× immersion oil) in: **(B)** a hypertrophied glomerulus with thickening of Bowman’s capsule is observed (arrow heads). Surrounding tubules show markedly thickened and wrinkled tubular basement membranes, and partial disruption of brush border (arrows). Interstitial expansion and hypercellularity around the glomerulus and surrounding tubules are also observed. These effects were markedly attenuated by l-cit treatment in diabetic mice. **(C)** Marked dilatation of cortical tubules (*), focal blebbing of the luminal edge (arrow), and focal detachment (arrow head) of epithelial cells in a proximal tubule.

Histological examinations of PAS-stained kidney sections of untreated diabetic WT mice at 16 weeks revealed glomerular hypertrophy, Bowman’s capsule thickening and peri-glomerulosclerosis, in comparison to control mouse kidneys (Figures 4A,B). The PT showed hypertrophy and markedly thickened and wrinkled basement membranes. Interstitial expansion and focal areas of hypercellularity were also observed. Treatment of diabetic WT mice with l-cit markedly ameliorated all diabetes-induced alterations in the kidney. Intriguingly, we observed a marked dilatation of cortical tubules, focal blebbing of the luminal edge of the cells and detachment in the kidneys from diabetic ArgII knock out mice (Figures 4A,C). However, no thickening of tubular basement membrane was observed in this group.

**Figure 5:**
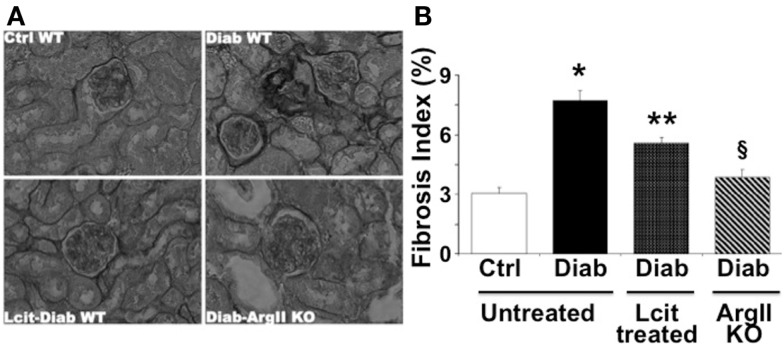
**Picro-sirius red staining of paraffin-embedded kidney sections**. **(A)** Representative photomicrographs of picro-sirius red-stained kidney sections from untreated control wild type (Ctrl WT), untreated diabetic wild type (Diab WT), l-cit-treated diabetic wild type (Lcit-Diab WT), and diabetic ArgII knock out (Diab-ArgII KO) mice at 16 weeks (RGB image using *Image J*). Increased collagen deposits (dark color) are observed in Diab WT. This effect was significantly reduced in Lcit-Diab WT, and in Diab-ArgII KO mice. **(B)** Fibrosis index (%) of tissue collagen content that indicates the ratio of the mean picro-sirius red-stained area to the mean whole area of the section. Values are mean ± SEM. *p < 0.001 vs. untreated Ctrl WT, **p < 0.01 vs. untreated Ctrl and Diab WT, ^§^*p* < 0.001 vs. untreated Diab WT.

As visualized in Figure 5A, picro-sirius red staining showed an enhancement of peri-glomerular and peritubular-interstitial collagen deposits in kidneys of WT diabetic mice at 16 weeks, as compared to control mice. This effect was reduced in l-cit-supplemented WT diabetic mice (Figures 5A,B). Interestingly, induction of diabetes by STZ in ArgII knock out mice did not result in enhanced collagen deposits, as compared to diabetic WT mice (Figures 5A,B).

### Anti-inflammatory effect of l-cit in diabetic mice

**Figure 6:**
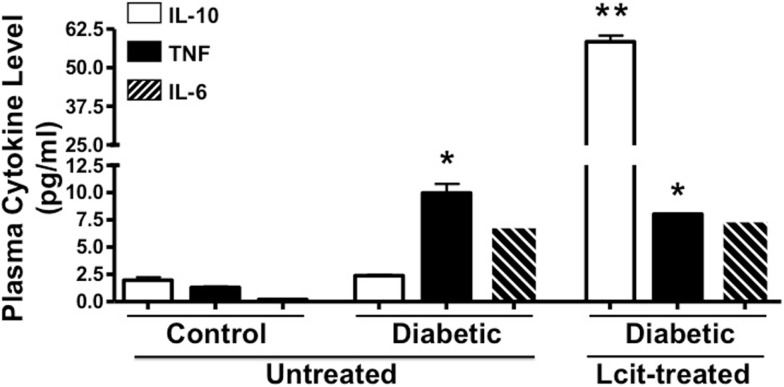
**Plasma cytokine level**. Mouse plasma samples were used for cytokine and chemokine measurement, using a magnetic bead-based multiplex assay. l-Cit induced an anti-inflammatory response in diabetic mice, as observed by significant increased levels of IL-10. Values are mean ± SEM. *p < 0.001 vs. untreated control mice, **p < 0.001 vs. untreated control and diabetic mice.

Because diabetes is considered a chronic inflammatory state (30), we examined the effect of l-cit supplementation on plasma cytokine levels in diabetic mice at the end of the experiment. We found enhanced levels of the pro-inflammatory cytokines TNF and IL-6 in the diabetic animals, with the former being significantly different when compared to control mice (Figure 6). Strikingly, the level of the anti-inflammatory cytokine IL-10 was significantly enhanced in plasma of l-cit-treated diabetic mice (Figure 6). We also found significantly enhanced levels of the pro-inflammatory chemokine MIP-2 in diabetic vs. control mice (ctrl: 0.2 + 0.02 pg/ml; STZ: 328.7 ± 2.6 pg/ml, *n* = *3*, *p* < 0.001 vs. ctrl). However, there was a significant reduction of MIP-2 upon l-cit supplementation to diabetic mice (l-cit/STZ: 311.6 ± 3.1 pg/ml, *n* = *3*, *p* < 0.05 vs. STZ, *p* < 0.001 vs. ctrl). These results thus indicate that l-cit treatment increases the anti-inflammatory response in STZ-treated diabetic mice.

### Effects of l-cit in diabetic STZ-rats

**Figure 7:**
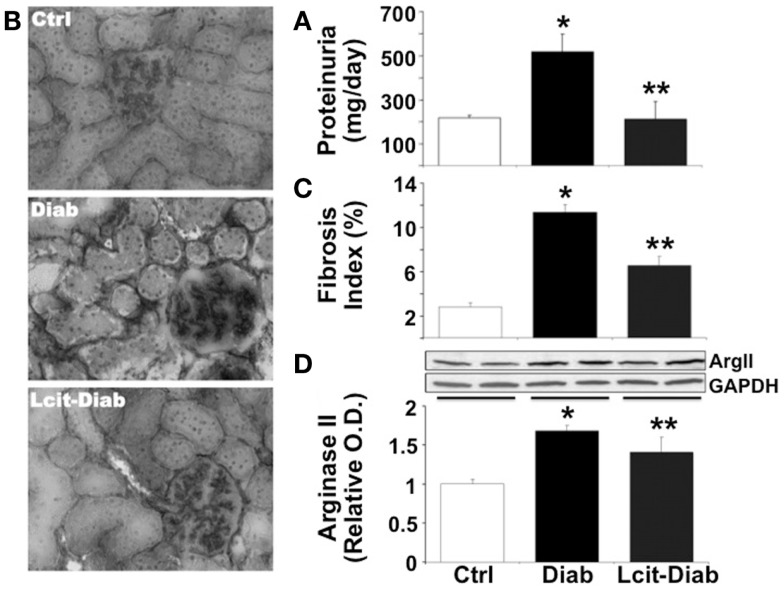
**Effect of l-cit on kidney pathology in STZ-rats**. Urine and renal tissues from untreated control (Ctrl), untreated diabetic (Diab), and l-cit-treated diabetic (Lcit-Diab) rats were evaluated. **(A)** Rats were placed in metabolic cages for 24 h urine collection. Total protein excretion was measured in urine. Lcit-Diab rats had a significant reduction of proteinuria when compared to Diab rats. *p < 0.05 vs. Ctrl, **p < 0.05 vs. Diab. **(B)** Representative photomicrographs of frozen sections from rat kidneys stained with picro-sirius red, show enhanced collagen deposits (dark color, RGB image using *Image J*) in untreated diabetic rats. This effect was markedly blunted in l-cit-treated diabetic rats. **(C)** Fibrosis index (%) of tissue collagen content that indicates the ratio of the mean picro-sirius red-stained area to the mean whole area of the section. *p < 0.001 vs. Ctrl, **p < 0.001 vs. Diab. **(D)** Renal ArgII protein expression determined by densitometric analysis of immunoblots performed with protein extracts from rat kidney cortex (*n* = 4, representative blot is shown). *p < 0.001 vs. Ctrl rats, **p < 0.05 vs. Ctrl rats.

Since C57BL6 mice develop only a moderate nephropathy upon STZ-treatment, we have also evaluated the effect of l-cit treatment in a more sensitive rodent model of STZ-induced diabetes, i.e., the rat. STZ-diabetic rats had increased daily proteinuria, as compared to non-diabetic control rats. However, l-cit treatment prevented this effect (Figure 7A). In addition, kidneys from untreated diabetic STZ-rats showed characteristic features of human DN, as observed by substantial collagen deposits of intraglomerular and peritubular distribution (Figures 7B,C). These effects were reduced in l-cit-supplemented STZ-rats (Figures 7B,C). These findings were accompanied by an elevation of renal ArgII protein levels in both, untreated and l-cit-treated diabetic STZ-rats, when compared to control non-diabetic rats (Figure 7D).

### Renal expression of kidney injury molecule 1

**Figure 8:**
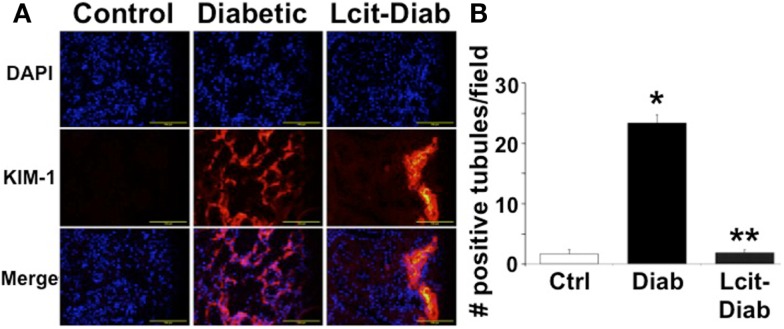
**KIM-1 immunofluorescence staining**. Kidney injury molecule 1 (KIM-1) antibody was used for immunofluorescent staining (red) of frozen sections from control (Ctrl), untreated diabetic (Diab), and l-cit-treated diabetic (Lcit-Diab) rats. Nuclei were counterstained with DAPI (blue). **(A)** Representative photomicrographs show negative staining for KIM-1 in control kidneys. Conversely, an increased number of tubules positively stained for KIM-1 is observed in kidneys of untreated diabetic rats. The number of positive tubules was dramatically decreased in kidneys of l-cit-treated diabetic rats. **(B)** Staining score determined by counting the number of positive tubules per field, using 40× magnification lens. Five to seven fields were examined in each kidney section (*n* = 3–4). *p < 0.001 vs. Ctrl, **p < 0.001 vs. Diab.

Kidney injury molecule 1 is a relevant biomarker of renal tubular damage that has been found to be associated with albuminuria in the early stage of nephropathy in diabetic patients (31), and with the progression of DN in experimental models (32). Therefore, we evaluated the expression of KIM-1 in kidneys of diabetic rats with or without l-cit supplementation. While renal tissues of control non-diabetic rats were negative for KIM-1 immuno-staining, numerous tubular segments in the cortex and in the outer strip of the outer medulla were intensely stained in diabetic rats (Figure 8A). l-Cit-treated diabetic rats showed fewer positive tubules than untreated diabetic rats. An objective score of the number of positive tubules per field is shown in Figure 8B.

### Effect of l-cit in human glomerular endothelial cells exposed to high glucose

**Figure 9:**
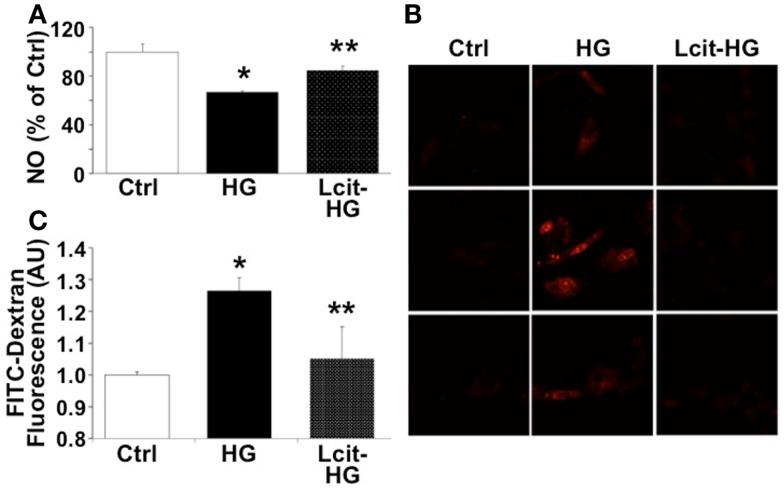
**Effect of l-cit on human glomerular endothelial cells exposed to high glucose**. HGECs were cultured in basic CSC medium containing 25 mM d-glucose (HG) with or without 1 M l-cit for 24 h **(A,B)** or 14 days **(C)**. l-Glucose (19.5 mM) was added to 5.5 mM d-glucose present in basic CSC medium, and used as iso-osmotic control (Ctrl). **(A)** The nitrite level (picomole) in the medium was determined by NO analyzer and expressed as percent of control (*p < 0.01 vs. Ctrl, **p < 0.05 vs. HG, *n* = 3). **(B)** HGECs were seeded onto gelatin-coated slide chambers. After completion of treatments, MitoSOX (a marker of mitochondrial superoxide, 5.0 μM) was added to the cells and incubated further for 10 min. Cells were washed and used for confocal microscopy imaging. Representative images show increase in mitochondrial MitoSOX fluorescence following treatment with HG. This effect was markedly reduced by concurrent l-cit treatment. **(C)** Transendothelial passage of FITC-dextran was used to determine permeability of HGEC monolayer, seeded onto collagen-coated Transwells. Fluorescent density of samples was analyzed on a paradigm microplate fluorometer (*p < 0.01 vs. Ctrl, **p < 0.05 vs. HG,*n* = 4).

Since HG-induced reactive oxygen species (ROS) generation is known to impair endothelial-derived NO production, we evaluated the effect of l-cit on NO production in HGECs exposed to HG. l-Cit pre-treatment of HGECs prevented the impaired NO production observed under exposure to HG for 24 h (Figure 9A). This effect correlated with a marked attenuation of mitochondrial superoxide generation, as opposed to the increase in mitochondrial red fluorescence intensity of MitoSOX in confocal microscopic images observed in HG-treated cells (Figure 9B).

Increasing evidence suggests that a NO/ROS imbalance causes endothelial barrier dysfunction (11). We therefore examined the effect of l-cit on HG-induced loss of barrier function in HGEC monolayers, by means of assessing their permeability to FITC-dextran. As shown in Figure 9C, HG (25 mM) significantly increased permeability of HGEC monolayers to FITC-dextran, but pre-treatment of the monolayers with l-cit (1 mM) conferred a significant protection from HG-induced hyperpermeability. These results suggest that l-cit protects glomerular barrier function at least in part by preserving glomerular endothelial NO synthase (NOS) function, and by reducing ROS generation under hyperglycemic insult.

### Effect of l-cit in human proximal tubular epithelial cells exposed to high glucose

**Figure 10:**
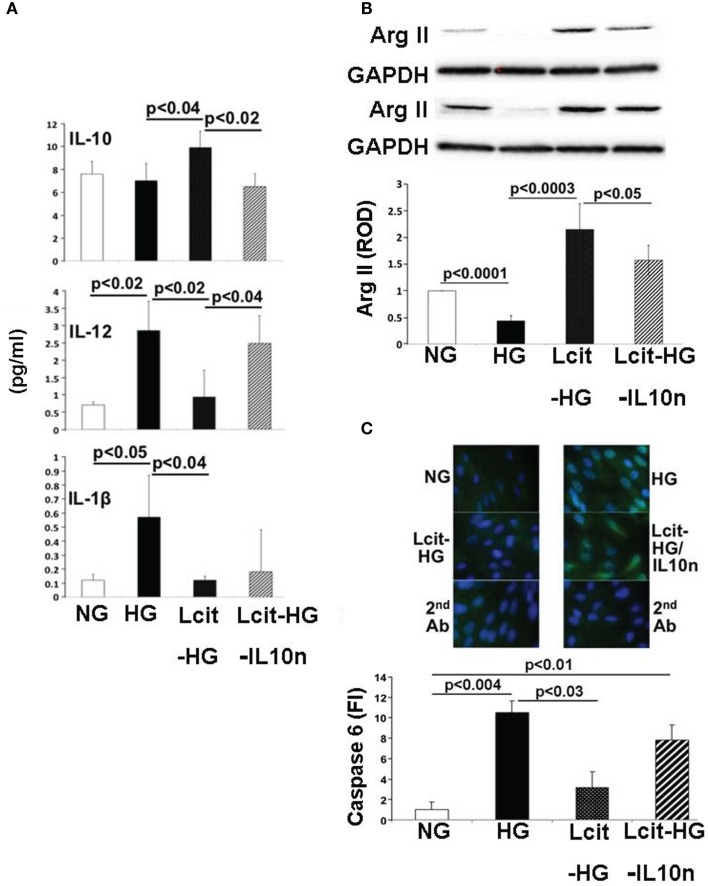
**Effect of l-cit in human proximal tubular epithelial cells (huPTEC) exposed to high glucose (HG)**. huPTEC were cultured in Renalife medium containing 25 mM d-glucose (HG) for 7 days. l-Glucose (17.8 mM) was added to 7.2 mM d-glucose present in medium, and used as iso-osmotic control (NG). Pre-treatment of huPTEC with l-citrulline (l-cit, 1 mM) was performed by adding the amino acid 2 h prior to adding HG or iso-osmotic control, with or without a neutralizing anti-IL-10 antibody (IL10n, 5 μg/ml). **(A)** The cytokine level (picogram per milliliter) in the medium was determined by using a commercial magnetic beads-based human cytokine kit. **(B)** Representative blot (upper) and densitometric analysis of blots (lower) show arginase II levels from protein extracts of huPTEC. A decreased in arginase II expression is observed following treatment with HG. This effect was markedly reduced by concurrent l-cit treatment, while addition of anti-IL-10 antibody (IL10n) along with l-cit partially prevented l-cit-induced elevation of arginase II. **(C)** huPTEC were seeded onto slide chambers. After completion of treatments, cells were immunostained using caspase 6 as primary antibody, followed by Cy5-conjugated goat anti-rabbit secondary antibody. DAPI was used for nuclear staining. Representative images (upper) and microscopy analysis of nuclear fluorescence intensity (lower) show an increase nuclear localization of caspase 6 (green fluorescence) following treatment with HG. This effect was markedly reduced by concurrent l-cit treatment, while the effect was abolished by addition of neutralizing anti-IL-10 antibody (IL10n) along with l-cit.

Proximal tubular cells are capable of generating IL-10 (33). Therefore, we investigated the effect of l-cit on cytokine production in huPTECs exposed to HG. huPTEC cultured under HG-supplemented medium in the presence of l-cit for 1 week, produced significantly enhanced levels of the anti-inflammatory cytokine IL-10, as compared to cells treated with HG alone (Figure 10A). This effect was accompanied by a significant reduction of levels of the pro-inflammatory cytokines IL-12 (p70) and IL-1β, the generation of which is increased in cells cultured under HG-supplemented medium without l-cit co-treatment. Addition of a neutralizing antibody against IL-10 to huPTEC cultured under HG in the presence of l-cit, significantly abolished the reduction of IL-12 (p70). In addition, elevation of IL-10 was accompanied by significant elevated protein levels of ArgII, an effect that was partially reduced when anti-IL-10 antibody was added along with l-cit to the HG-supplemented medium (Figure 10B). These data indicate that elevation of ArgII in huPTEC in culture is a marker of the anti-inflammatory actions of l-cit through its IL-10-inducing capacity.

We also examined the activation of the apoptotic marker caspase 6, which was shown to be involved in PTEC apoptosis during nephropathy (34). We observed an increased nuclear translocation of caspase 6 in huPTEC exposed to HG. l-Cit significantly blunted this effect of HG at least partially in an IL-10-dependent manner, since concurrent treatment of huPTEC with a neutralizing IL-10 significantly prevented the reduction in caspase 6 nuclear translocation by l-cit (Figure 10C). This indicates that the observed caspase 6 activation was linked to a pro-inflammatory cytokine, the generation of which was inhibited by IL-10.

## Discussion

Hyperglycemia, which activates several reactions, including oxidative stress and chronic or subclinical inflammation, is clearly recognized as the primary player in diabetic endothelial dysfunction and DN (35–37).

It is now generally recognized that an important cause of impaired endothelial NO production, characteristic of diabetic endothelial dysfunction, is reduced availability of the eNOS substrate l-arg. Despite diverse data from studies assessing plasma amino acid levels in diabetic patients with or without chronic kidney disease (38, 39), patients with diabetes and cardiovascular disease were shown to benefit only from acute (2), but not from chronic (3) l-arg supplementation.

Conversely, we and others have been shown that oral l-cit (precursor of l-arg) augments NO-dependent signaling, not only by means of increasing l-arg synthesis, but also by decreasing l-arg catabolism, as such increasing circulating l-arg levels (4–6). However, the effects of l-cit on the development of diabetic kidney damage have not been studied. Therefore, in this study, we assessed the actions of supplemental l-cit in a murine model of DN. Our data demonstrate that oral l-cit supplementation protects diabetic STZ-mice from the sustained elevation of UAE, as observed in untreated mice at 16 weeks of the disease. This protective effect of l-cit occurs despite significant hyperglycemia. We also observed similar benefits conferred by l-cit in a more aggressive model of DN in STZ-rats, which also showed reduced proteinuria after 8 weeks of treatment.

Diabetic urinary albumin leakage involves several mechanisms, including proximal tubular injury (40, 41) and disruption of the glomerular barrier (42). The relevance of the glomerular endothelium in the maintenance of barrier function has only been recently recognized (12). While endothelial NO generation contributes to endothelial glycocalyx and barrier preservation (11, 43, 44), an increase in the ROS/NO ratio causes disruption of the glycocalyx, resulting in enhanced albumin permeability (45, 46).

We have found a reduction in mitochondrial ROS generation, combined with a restoration of NO production in HGECs treated with l-cit before exposure to HG-supplemented medium. This effect may thus at least partially account for the reduced glomerular albumin leakage we have found in the diabetic animals supplemented with l-cit. In support of this is the reduced permeability to FITC-dextran of HGEC monolayers exposed to HG and concurrently treated with l-cit.

We did not assess either constitutive (endothelial and neuronal) or inducible NOS expression in the kidneys of our diabetic animals, because the three NOS isoforms have been described to be differentially altered in DN (47, 48). Indeed, the discrepant results on NOS expression and NO involvement in diabetic pathology have been evaluated in other diabetic complications (49, 50).

Our current results, along with previous work, support the notion that bioavailability of NO is reduced in the diabetic vessels (8, 51, 52). Therefore, adding l-cit to current therapies may lead to a safe and efficacious option to improve vascular diabetic complications. Moreover, due to the significant role of NO in the regulation of insulin release from pancreatic β-cells (53), l-cit may also be useful as a potential insulinotropic agent. However, the effect of l-cit on pancreatic β-cell function requires further studies.

Several studies have also suggested a role for endothelial NO in suppressing fibrotic pathways in different organs and pathologies associated with diabetes and other diseases (54, 55). As such, the protective effect of l-cit on eNOS function may have led to the reduction in kidney fibrosis, as observed in our study in diabetic mice and rats after 16 and 8 weeks of diabetes, respectively.

The protective effects of l-cit toward UAE and kidney fibrosis were observed despite a sustained elevation of ArgII protein levels in the renal cortex. ArgII protein was significantly elevated in l-cit treated diabetic mice and rats over control and untreated diabetic animals at the end of the study, when protection on kidney pathology was more evident. Interestingly, the protective effects of l-cit administration in other pathologies have also been shown to be accompanied by an enhanced tissue expression of ArgII (23).

Our findings prompt the question about the role of ArgII for tubular function in diabetes. ArgII is present in the mitochondria of PT, as well as of inner medullary collecting ducts (17) and provides l-ornithine for the synthesis of polyamines (56). The cellular balance of polyamines is necessary for DNA stabilization and replication (57), as well as for the maintenance of PT integrity and function (58).

Damage of PT under the insult of HG levels, especially in patients with poor glycemic control (59), requires an extensive repair process, either by regeneration of de-differentiated surviving cells (60) or by proliferation and differentiation of stem cells (61). It has been recently demonstrated that spermidine enhances epithelial stem cell function (62). Thus, adequate polyamine levels may allow the PT to resume normal functions, and l-cit may facilitate this process by providing more l-arg for ArgII function.

In addition, up-regulation of mitochondrial ArgII in diabetic PT may represent a stress response to an increased energy demand in this actively reabsorptive segment of the nephron. Arginase-derived ornithine in the mitochondria may be converted to l-glutamate that enters the tricarboxylic acid cycle as oxoglutarate (63). l-cit could as such provide precursors to maintain the energetic metabolism of PT via mitochondrial ArgII. Diabetic kidneys from l-cit-treated rats clearly showed a reduced number of positive tubules for KIM-1 expression, a marker of proximal tubular damage. This effect may also be associated with an improvement of proteinuria (64) observed in l-cit treated diabetic rats.

The results of our comparative studies between WT and ArgII knock out mice partially differ with a recent report by others (22). Despite a trend to reduced levels of albuminuria in diabetic ArgII knock out mice, the reduction was not significantly different from untreated WT diabetic mice. Differences between the Morris study and ours likely arise from the significantly greater age of our mice. Indeed, we observed a severe dilation and morphological alterations of cortical tubules, as well as greater BUN levels in ArgII knock out mice, as compared to untreated diabetic WT mice. These findings suggest that with advanced age, lack of ArgII may limit tubular repair and may accelerate the decay in glomerular filtration rate observed in the diabetic condition. Other reported mechanisms may also apply for tubular damage in ArgII knock out mice (65, 66). To that purpose, it would be interesting to determine in future studies, whether l-cit supplementation to ArgII knock out mice prevents diabetes-induced tubular damage and enhancement of BUN levels.

Intriguingly, collagen deposition in kidneys isolated from diabetic ArgII knock out mice was not different from the one observed in control WT mice. A limited availability of the precursor proline, provided by the ArgII/ornithine aminotransferase pathway, may be the cause of reduced renal collagen synthesis/crosslink in this group, which indicates that ArgII has an important contribution to renal collagen content. However, the cost-effect of specific ArgII inhibition in advanced stages of diabetic animal models remains to be investigated.

In addition to the previous findings, l-cit treatment to diabetic mice prevented body wasting even in the absence of blood glycemic control. Type 1 diabetic patients under poor glycemic control, common in low income or un-insured patients in the United States or in under-developed countries (67) exhibit detrimentally low intracellular energy metabolism and significant weight loss, leading to chronic fatigue and general body weakness. l-Cit may protect against diabetic muscle wasting via nutritional support, providing the precursor for creatine synthesis (68, 69).

A substantial benefit conferred by l-cit supplementation is the significant elevation of the anti-inflammatory cytokine IL-10. It has been recently recognized that common inflammatory factors play a role in both type 1 and 2 diabetic pathology (30, 70), which has important therapeutic implications (71–73). IL-10 has been shown to selectively induce ArgII expression in macrophages (74) and to also attenuate a pro-inflammatory cytokine expression and iNOS-derived NO production in human and mouse monocyte/macrophage cells in the presence of apoptotic cells (75). Apoptosis of PT epithelial cells is a feature of the hyperglycemic insult in DN, and activated PT epithelial cells, as an alternative to macrophages, are able to phagocytose neighboring apoptotic cells (76). Moreover, PT epithelial cells play an important role in anti-inflammatory mechanisms within the tubulointerstitium during renal injuries (77, 78) and are capable of generating their own production of IL-10 (33).

Although we did not measure local kidney tissue or urinary levels of IL-10 in our study, but rather in plasma, it is nevertheless likely that both local and systemic anti-inflammatory mechanisms may take place under the setting of l-cit supplementation, since this treatment was accompanied by enhanced ArgII expression in PT, and by increased plasma levels of IL-10. In support of our findings *in vivo*, we observed that huPTECs cultured under HG-supplemented medium in the presence of l-cit for 1 week, produced significantly enhanced levels of the anti-inflammatory cytokine IL-10, as compared to cells treated with HG alone. This is a prominent feature of l-cit’s actions on huPTEC, which may be of high significance in the context of current clinical trials aimed to limit inflammation in diabetic patients, and to reduce progression of DN toward ESRD. In correspondence with our observations that l-cit increases IL-10 generation, it has been recently shown that *Citrullus lanatus* (Watermelon), a rich source of l-cit, was beneficial in a murine inflammatory disease model, by means of increasing plasma levels of IL-10 (79).

The enhanced production of the anti-inflammatory cytokine IL-10 by l-cit in HG-treated huPTEC was accompanied by a reduction of levels of the pro-inflammatory cytokines IL-12 (p70) and IL-1β both of which were induced above basal levels by HG in cells not treated with l-cit. *In vivo*, these pro-inflammatory cytokines may establish the settings for a crosstalk between tubular cells and surrounding infiltrating leukocytes, to amplify the inflammatory milieu of diabetic kidneys. As such, l-cit-induced IL-10 generation may be important in limiting inflammation in the kidney.

In accordance with our findings on ArgII expression in kidneys of mice at late stages of diabetes, cultured huPTEC under HG condition had a reduced expression of ArgII protein levels. l-Cit significantly enhanced ArgII in HG-treated huPTEC, an effect that was reduced by co-administration of IL-10 neutralizing antibody with l-cit. These results indicate that up-regulation of ArgII in PT is a marker of the anti-inflammatory actions of IL-10 on renal tubules.

We could detect an increased level of nuclear localization of the executioner caspase 6, a mediator of apoptosis, in huPTEC exposed to HG levels. l-Cit blunted this effect of HG at least partially in an IL-10-dependent manner. This indicates that the observed caspase 6 activation was linked to a pro-inflammatory cytokine, the generation of which was inhibited by IL-10. Although more research is needed to unravel what cytokines are responsible for the caspase 6 activation in HG-treated huPTEC, an interesting candidate could be IL-1, which was shown to be increased by HG in huPTEC in our experiments and whose generation was blunted by l-cit. IL-1 was shown to induce Fas ligand generation, a potent inducer of apoptosis in renal tubular cells (80).

In conclusion, our study demonstrates that l-cit supplementation is protective to the nephron function. l-Cit not only reduces UAE and prevents collagen deposits in the kidneys of diabetic animals, but also establishes the settings for an anti-inflammatory response in the PT, with the potential to direct the immune response toward an anti-inflammatory profile in monocyte/macrophages as well. These observations are substantiated by the elevation of tubular ArgII expression, and of plasma levels of IL-10.

It remains to be established whether l-cit sustains tubular mitochondrial function, by providing precursors *via* ArgII and whether this effect is linked to repair processes of the proximal nephron under the hyperglycemic insult. As such, this work lays the foundation for a broader investigation of the effects of l-cit supplementation on local vs. systemic IL-10 generation, which may have important therapeutic applicability in diabetic patients.

## Conflict of Interest Statement

The authors declare that the research was conducted in the absence of any commercial or financial relationships that could be construed as a potential conflict of interest.
